# Epidemiological and clinical burden associated with plexiform neurofibromas in pediatric neurofibromatosis type-1 (NF-1): a systematic literature review

**DOI:** 10.1007/s10072-021-05361-5

**Published:** 2021-06-18

**Authors:** Ike Iheanacho, Hyun Kyoo Yoo, Xiaoqin Yang, Sophie Dodman, Rachel Hughes, Suvina Amin

**Affiliations:** 1 Evidence Synthesis, Modeling and Communication, Evidera, The Ark, 201 Talgarth Rd, London, W6 8BJ UK; 2 AstraZeneca, Gaithersburg, MD 20871 USA; 3 Merck & Co., Inc, Kenilworth, NJ USA

**Keywords:** Neurofibromatosis type-1; Plexiform neurofibroma, Pediatric, Burden, Epidemiology

## Abstract

**Purpose:**

Patients with neurofibromatosis type-1 (NF-1) and associated plexiform neurofibromas (PNs) often have a high burden of illness owing to debilitating symptoms of these tumors and limited management options. To investigate this complex disease, a systematic literature review (SLR) was conducted on the epidemiology of pediatric NF-1 and associated PNs, the burden of illness, and outcomes of surgical resection of these tumors.

**Methods:**

Searches of MEDLINE and Embase (from database inception to October 2019) and conference proceedings (2017–2019) were performed to identify relevant studies. The review methodology was informed by the Preferred Reporting Items for Systematic Reviews and Meta-Analyses guidelines.

**Results:**

Twenty studies were identified. Evidence confirmed NF-1 is rare but that occurrence may differ geographically. Only limited data on the birth incidence of NF-1 were identified. Prevalence estimates for pediatric NF-1 varied from one per 960 individuals (aged 17 years) to one per 5681 children (aged < 16 years) across five large registry/surveillance studies (each involving > 19,000 individuals). The prevalence of associated PNs was 0–29.6%. PNs carried increased mortality risk in pediatric NF-1 in both studies that explored this potential association. Patients with PNs reported high use of analgesics. The complication rate post-surgery for PNs was around 17–19%. The recurrence rate (18–68%) was dependent on the extent of excision achieved during surgery.

**Conclusions:**

Data suggest NF-1 is a rare disease with increased morbidity and mortality in children with associated PNs. Surgical outcomes for PNs are often poor. These findings suggest significant unmet needs in patients with NF-1-associated PNs.

**Supplementary Information:**

The online version contains supplementary material available at 10.1007/s10072-021-05361-5.

## Introduction

Neurofibromatosis type-1 (NF-1) is an autosomal-dominant genetic disease (caused by sporadic mutations in 50% of cases [1]) characterized by the development of multisystem tumors [2], particularly neurofibromas—benign nerve sheath tumors that can cause pruritus, pain, sensory impairment, and motor dysfunction [3]. Other NF-1 manifestations include abnormal skin pigmentation, iris Lisch nodules, skeletal abnormalities, cardiovascular complications, and learning difficulties [3, 4]. Typically, these features begin in early childhood. For example, one study found the proportion of sporadic NF-1 cases that met the National Institutes of Health (NIH) Diagnostic Criteria was around one-half by 1 year of age and 97% by 8 years [2].

Individuals with NF-1 are also at an increased risk of developing other tumors [3, 4]. In particular, an estimated 30–50% develop plexiform neurofibromas (PNs) [3], which involve multiple nerve fascicles and can transverse the length of nerves. These lesions can cause pain, disfigurement, bone destruction, and compression of vital structures [5], features that begin in early childhood and progress throughout life [6]. In addition, PNs may undergo transformation to malignant nerve sheath tumors (MPNST) [3]. Estimates suggest that 8–13% of NF-1 patients develop such lesions during their lifetime, although some of these tumors occur in the absence of pre-existing PNs [7].

Current management guidelines for NF-1 and PNs focus on continuously monitoring patients for the development or progression of clinical features, such as pain [2, 3]. For tumor progression, surgical resection is considered the standard of care. However, surgery carries risks, may not offer definitive treatment (e.g., if complete excision is impossible), and may be followed by recurrence [2, 3, 8].

Consequently, patients with NF-1-associated PNs may have considerable unmet needs, related to disabling symptomatology and limited management options. Understanding and addressing any such needs in children is crucial, given NF-1 is a developmental and progressive disorder, with morbidity and mortality that may differ across age groups. However, there is a lack of published, collated evidence characterizing various aspects of this disease burden. Targeting these data gaps, this systematic literature review (SLR) aimed to synthesize published evidence on epidemiological outcomes for pediatric NF-1 and NF-1-associated PNs, on pain related to PNs, and on outcomes of surgery to resect or debulk PNs.

## Methods

The methodology for this SLR was informed by standards in the Preferred Reporting Items for Systematic Reviews and Meta-Analyses (PRISMA) guidelines [9].

### Literature searches and data sources

Searches were conducted in Embase and MEDLINE via OvidSP in October 2019 using a strategy that combined terms (free-text keywords and controlled subject headings) for relevant populations (patients with NF-1/NF-1-associated PNs) with terms for the topics of interest (epidemiology; pain; surgery), without publication date limits. Further details on the searches are presented in the Online Resource. Also, recent proceedings (from 2017–2019) of selected conferences were searched for relevant evidence that had not yet been published in peer-reviewed journals.

### Study selection

Studies were screened for inclusion against the pre-defined population, interventions, comparators, outcomes, and study design (PICOS) criteria in Table 1. To be eligible, studies had to have (1) investigated epidemiological outcomes in children (aged ≤ 18 years) with NF-1, and/or (2) evaluated pain or surgical outcomes associated with PNs in pediatric NF-1. No geographic or temporal exclusion criteria were applied. All abstracts and full-text articles were screened by two independent investigators, with any conflicts resolved by a third investigator.

**Table 1:** PICOS criteria

Domain	Epidemiology	Burden of pain	Burden of surgery
Population	•Pediatric patients (aged ≤ 18 years)^a^ with NF-1	• Pediatric patients (aged ≤ 18 years)^a^ with NF-1	• Pediatric patients (aged ≤ 18 years)^a^ with NF-1
Intervention	• None required	• None required	• Surgical interventions
Comparators	• None required	• Any or none required	• Any or none required
Outcomes	• Incidence • Prevalence • Mortality	• Incidence of pain • Prevalence of pain • Pain intensity • Functional outcomes and mobility outcomes related to pain • Real-world treatment outcomes for pain • Humanistic outcomes in pain (including HRQoL, effects on sleep, and psychological outcomes) • Economic outcomes (healthcare resource use and costs) associated with pain	• Indication for surgery • Operation rates • Complete and partial resection rates • Tumor recurrence rate following surgery • Secondary surgery rates • Bleeding complications • Wound healing complications • Post-operative HRQoL • Neurological complications
Study design	• Retrospective and prospective, observational studies • Cross-sectional studies • SLRs^b^	• Clinical trials • Retrospective and prospective, observational studies • Cross-sectional studies • SLRs^b^	• Clinical trials • Retrospective and prospective, observational studies • Cross-sectional studies • SLRs^b^
Search limits			
Publication date	No time limit specified		
Publication type	Full-text publications and conference abstracts		
Geographical region	No limit		
Language	No limit in searches, but only English-language publications are included in the review		

^a^Studies that enrolled adults and children with NF-1 were eligible for inclusion if the study reported separate subgroup data for pediatric patients

^b^Bibliographies of relevant SLRs/meta-analyses identified by the database searches were hand-searched for any additional, potentially relevant studies

Abbreviations: HRQoL = health-related quality of life; NF-1 = neurofibromatosis type 1; PICOS = population, interventions, comparators, outcome, study design; PN = plexiform neurofibroma; SLR = systematic literature review

### Data extraction and synthesis

Data were extracted by one investigator and then validated by a second investigator. A third investigator was consulted to resolve any disagreements. For added quality assurance, final checks of all extracted data were conducted, to ensure consistency in how this information was captured from publications.

Results were collated and assessed by using qualitative synthesis. Specifically, studies were grouped according to key themes identified, and the connections between studies and the objectives of the review were noted, interpreted, and summarized accordingly. No formal risk-of-bias assessment was conducted because the disparate nature of study designs included in the review precluded the use of a single standard recognized tool for such analysis. However, study sample sizes and data sources were considered as broad determinants of study quality, under the assumption that larger, population-based or multicenter studies were more likely to provide robust and generalizable results than were smaller studies.

## Results

### Study selection

**Fig. 1:**
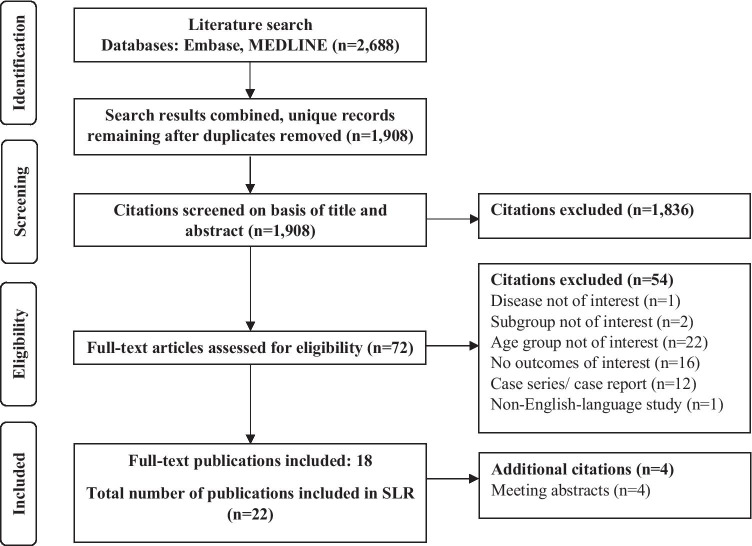
PRISMA diagram. Abbreviations: PRISMA, Preferred Reporting Items for Systematic Reviews and Meta-Analyses; SLR, systematic literature review

The database searches yielded 2688 articles, 18 of which met the study selection criteria. Another four articles were identified through supplementary searches, resulting in 22 included publications on 20 unique studies (Fig. 1).

### Overview of study characteristics

**Fig. 2:**
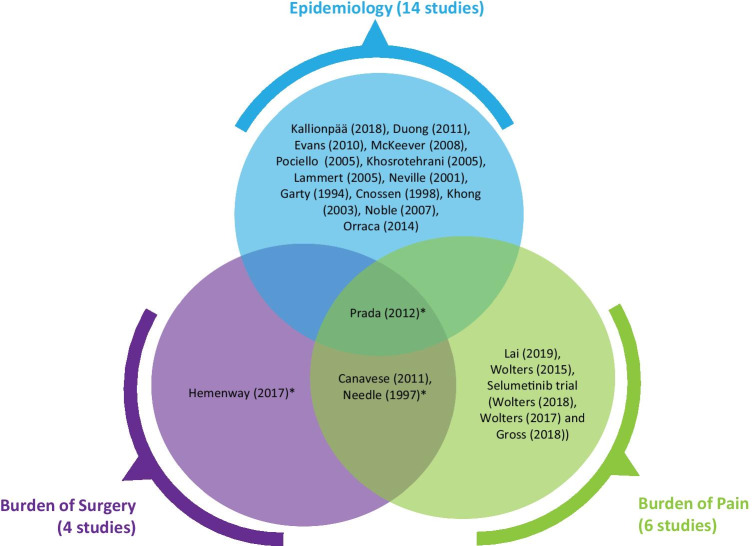
Overview of the outcomes reported in included studies. *Studies report tumor recurrence

Of the 22 publications identified, 14 (64%) reported on the epidemiology of NF-1 or PN in NF-1 [10–23]. One epidemiology study also reported on the burden of pain and outcomes for patients undergoing PN surgery (Fig. 2) [23]. In total, four studies reported on the burden of PN-related surgery [23–26], and six unique studies (eight publications) examined the burden of pain in this population.

Two epidemiology studies were conducted in the United Kingdom (UK) [12, 18], two in the United States (US) [19, 23], and one in each of following locations: Australia [20], Cuba [21], Finland [14], France [11], Germany [17], Israel [13], Italy [22], the Netherlands [10], North America [10, 16], and Hong Kong [15]. All studies reporting on pain associated with PNs and outcomes of PN surgery were US based [23, 25–31] (Table 2).

**Table 2:** Study characteristics

Author, year	Study design	Data source	Country	Year(s) of study	Brief description of the study population	Sample size	Length of follow-up
Epidemiology studies							
Kallionpää R, 2018 [14]	Retrospective, nationwide, population-based prevalence study	Review of medical records from secondary and tertiary referral centers	Finland	Data collection: 1987–2011	All patients who fulfilled the NIH diagnostic criteria for NF-1	Finnish NF-1 cohort: 1476 patients Total surveillance population: NR	21,742 person-years (median: 15.0 years per person; range: 0.01–28.0 years per person)
Duong TA, 2011 [11]	Retrospective cohort study	NF-1 Network Database maintained by the National French Hospital Database (PMSI)	France	January 1980–December 2006	Cohort of consecutive NF-1 patients in France referred to hospital departments of the Paris conurbation that constitutes the National French Referral Centre for Neurofibromatosis	1895	Median: 6.8 years (range 0.4–20.6 years)
Evans DG, 2010 [12]	Retrospective analysis of cancer surveillance registry and genetic register	The North West Regional Cancer Intelligence Service and the North West Regional family GR	UK	1953–2003	NF-1 patients residing in the North West England, Greater Manchester area	Total population studied: 3,050,409	NA
McKeever K, 2008 [18]	Retrospective review of an NF-1 case registry	Department of Medical Genetics in Belfast City Hospital	UK (Northern Ireland)	1990–2002	All cases of NF-1 in children less than 16 years of age in Northern Ireland	The total population size in Northern Ireland at the time of the study was reported to be 1.68 million, of which 425,250 were under 16 years of age. Most children with NF-1 are referred to the Department of Medical Genetics in Belfast. 75 children with NF-1 were identified	NR
Pociello R, 2005 [22]	Prospective cohort study	Scholastic institutes of the XVII, XVIII, XX districts of Rome: Leopardi, Pistelli, Umberto I, Convitto Nazionale, Cairoli, Pianciani, Peroni, Woytila, XXI Aprile, Alfieri	Italy	Dates NR Recruitment: three years duration	Cohort of two groups of children (6 and 10 years) recruited through scholastic institutes of the XVII, XVIII, XX districts of Rome and evaluated for early detection of NF-1	Total population observed: 2513 Cases of NF-1: 6 Suspected cases: 201	16 years
Khosrotehrani K, 2005 [16]	Prospective cohort study	NFID	North America	1977–1996	Patients who fulfilled the NIH diagnostic criteria for NF-1	405	Mean, 2.4 years (range: 0–15.3 years)
Lammert M, 2005 [17]	Cross-sectional survey	Routine medical examinations at elementary school enrollment	Germany	2000 and 2001	Children aged 6 years old in six German states	Total surveillance population: 152,819	NA, cross-sectional survey
Neville H, 2001 [19]	Retrospective review of medical charts	Neurofibromatosis database established through the Neurofibromatosis Clinic at The University of Texas M.D. Anderson Cancer Center	US (Texas)	1979–1999	All children (< 21) with neurofibromatosis who underwent an operative procedure at the University of Texas-Houston Medical School, the Memorial-Hermann Children’s Hospital, and the University of Texas M.D. Anderson Cancer Center	249	NR
Garty BZ, 1994 [13]	Cross-sectional survey	Jewish recruits for military service	Israel	NR	17-year-old Jewish recruits for military service	Total surveillance population: 374,440	NA, cross-sectional survey
Cnossen MH, 1998 [10]	Prospective cohort study	Sophia Children’s University Hospital	Netherlands (Rotterdam)	1985–1995	Children (younger than 18 years of age) with a suspected diagnosis of NF-1	150	• Group A: mean ± SD duration of follow-up was 4.9 ± 3.8 years • Group B: total number of person-years in children presenting without complications: 340.8 •Group C: total number of person-years in children with one or two complications at presentation: 322.6
Khong PG, 2003 [15]	Prospective surveillance study	Tertiary referral center	Hong Kong	1995–2001	Consecutive children seen in an NF-1 clinic in a tertiary referral center	53	Follow-up imaging was performed in 10 children over a mean period of 29 months
Noble F, 2007 [20]	Retrospective review of genetic and hospital files	NF-1 clinic at the Royal Children’s Hospital in Melbourne	Australia (Melbourne)	2001–2004	Patients with confirmed or presumed diagnoses of NF-1 who attended NF-1 clinic at a tertiary hospital	121	NR
Orraca M, 2014 [21]	Cross-sectional survey	Children aged 9–11 years (i.e., born between January 1, 1993, and December 31, 1994) living in the province at the time	Cuba (14 municipalities in the province of Pinar del Río)	2004	Children aged 9–11 years (i.e., born between January 1, 1993, and December 31, 1994) living in 14 municipalities in the province of Pinar del Río, Cuba	Total population size: 19,404 Total population assessed for NF-1 in the survey: 19,392	NA, cross-sectional survey
Epidemiology, burden of pain and surgery							
Prada CE, 2012 [23]	Retrospective review of medical records	Cincinnati Children’s Hospital Medical center	US (Ohio, Indiana, and Kentucky)	1997–2007	Pediatric cohort of patients with NF-1 (with and without symptomatic PNs)	520 NF-1 with PNs: 154 NF-1 without PNs: 366	Average follow-up for NF-1 with PNs: 6.4 years (range, 4–10 years)
Burden of pain and surgery							
Canavese F, 2011 [26]	Retrospective review of medical charts and radiographs of consecutive cases of NF-1 among children and adolescents	Shriners Hospital Portland	US (Portland, Oregon)	1994–2006	Consecutive pediatric NF-1 patients with PN of the trunk or the extremities who underwent surgical resection of the tumor	100 consecutive NF-1 patients were assessed, 14 met inclusion criteria, 10 of whom were < 18 years old	Average: 65.5 months (range: 24–145 months)
Needle MN, 1997 [25]	Retrospective review of the inpatient and outpatient records	Department of Surgical Pathology reports (The Children’s Hospital of Philadelphia)	US (Philadelphia)	1974–1994	Children with NF-1 and PN who had undergone a surgical procedure	121	Median follow-up: 6.8 years (range 2 months to 24.5 years)
Burden of pain							
Lai J, 2019 [28]	Cross-sectional, non-interventional, patient-reported outcomes study	The Children’s Tumor Foundation NF Registry, Regional NF-1 organizations and The Ann & Robert H. Lurie Children’s Hospital of Chicago	US (Chicago)	NR	Children aged 8–17 years old with a confirmed diagnosis of NF-1 and who had at least one PN in any location (symptomatic or asymptomatic)	140	NA
Wolters PL, 2015 [31]	Cross-sectional analysis of a longitudinal study	NCI Natural History Study of Patients with NF-1	US	NR	Children and adolescents with NF-1 and PNs aged 6–18 years, enrolled in the NCI natural history study	60	NA
Wolters P, 2018 [30] Wolters P, 2017 [29] Gross AM, 2018 [27]	Single-arm trial	Phase II selumetinib trial (NCT 0136 2803)	US	Trial ongoing (2011–NA)	Children aged 2–18 years with NF-1, inoperable PNs, and PN-related morbidity	50	1 year
Burden of surgery							
Hemenway M, 2017 [24]	Retrospective cohort study	Children’s Hospital Colorado	US	NR	Patients with PN who met the criteria for surgical resection and were treated according to the Colorado Children’s Hospital treatment pathway	11	6 months

Abbreviations: *GR*, genetic register; *NA*, not applicable; *NCI*, National Cancer Institute; *NF-1*, neurofibromatosis type 1; *NFID*, Neurofibromatosis Institute Database; *NIH*, National Institutes of Health; *NR*, not reported; *PMSI*, Programme National de Médicalisation des Systèmes d’Information; *PN*, plexiform neurofibroma; *SD*, standard deviation; *UK*, United Kingdom; US, United States

### Epidemiology

Across the 14 studies providing data on the epidemiology of NF-1, there was only limited evidence (two studies) on the birth incidence of NF-1. Six studies presented prevalence estimates for NF-1 in general. Two studies compared mortality rates in NF-1 patients with and without associated PNs. Overall, the available global epidemiology evidence confirmed that NF-1 is a rare condition but with an occurrence that may differ markedly across countries [10–23]. It also showed that PNs are both common [10, 15, 16, 18, 20, 23], and potentially life-limiting in pediatric cases [16, 23] (Table 3).

**Table 3:** Epidemiology of NF-1 and PNs associated with NF-1

Author, year	Study dates	Country	Sample size	Age group	Incidence of NF-1	Prevalence of NF-1	Incidence of PNs in NF-1	Prevalence of PNs in NF-1
Cnossen, 1998 [10]	1985–1995	Netherlands (Rotterdam)	150	< 18 years	NR	NR	0.6 per 100 PY (n = 2)	40/150 (26.7%)
Evans, 2010 [12]	1953–2003	UK	3,050,409^a^	NR	Median birth incidence: 1/3657 (95% CI: NR) Maximum birth incidence: 1/2712 (95% CI: NR)	NR	NR	NR
Garty, 1994 [13]	NR	Israel	374,440	17-year-olds	NR	390/374,440 (95% CI: NR)	NR	NR
Kallionpää, 2018 [14]	2005	Finland	Total sample size: NR	0–4 years	NR	Estimated prevalence: 1/1706 (95% CI: 1/2158–1/1410)	NR	NR
				5–9 years	NR	Estimated prevalence: 1/1719 (95% CI: 1/2176–1/1421)	NR	NR
				10–14 years	NR	Estimated prevalence: 1/1731 (95% CI: 1/2192–1/1431)	NR	NR
				15–19 years	NR	Estimated prevalence: 1/1757 (95% CI: 1/2225–1/1451)	NR	NR
Khong, 2003 [15]	1995 and 2001	Hong Kong	53	11 months–18 years	NR	NR	NR	Spinal PNs: 3/53 (5.7%)
Khosrotehrani, 2005 [16]	1977–1996	North America	376	< 17 years	NR	NR	NR	Facial PNs: 34/376 (9.0%)
Lammert, 2005 [17]	2000–2001	Germany	152,819	6-year-olds	NR	1/2996 (95% CI: NR) 2000: 1/3072 2001: 1/2938	NR	NR
McKeever, 2008 [18]	1990–2002	Northern Ireland	425,250	< 16 years	NR	1/5681; 17.6 per 100,000 population (95% CI: NR)	NR	7/75 (9.3%)
Noble, 2007 [20]	2001–2004	Australia (Melbourne)	121	< 10 years	NR	NR	NR	7/68 (10.3%)
				10–20 years	NR	NR	NR	8/35 (22.9%)
Orraca, 2014 [21]	1993–1994	Cuba (14 municipalities in the province of Pinar del Río)	19,392	9–11 years	NR	1/1141; 87.7 per 100,000 (95% CI: NR) 1993: 1/1340 1994: 1/1001	NR	0/17(0%)
Porciello, 2005 [22]	NR	Italy	2,513	6- and 10-year-olds	Birth incidence of NF-1 1/400 (95% CI: NR)	NR	NR	NR
				6-year-olds	NR	5/1320 (95% CI: 0.12–0.88), p = 0.222	NR	NR
				10-year-olds	NR	1/1193 (95% CI: 0.002–0.47), p = 0.222	NR	NR
Prada, 2012 [23]	1997–2007	US (Ohio, Indiana, and Kentucky)	520	< 18 years	NR	NR	NR	154/520 (29.6%)

^a^Total number of births in a region between 1953 and 2003

Abbreviations: *CI*, confidence interval; *NF-1*, neurofibromatosis type 1; *NR*, not reported; *PN*, plexiform neurofibroma; *PY*, person-year; *UK*, United Kingdom; US, United States

One of the two studies reporting birth incidence estimates, a population-based registry analysis [12] in the UK (n = 3,050,409), found a median rate of one per 3657 people [12]. The second, much smaller, and therefore potentially less representative, Italian school-based surveillance study (n = 2513) estimated a birth incidence of one case per 400 births [22].

Estimates of the prevalence of NF-1 [13, 14, 17, 18, 21, 22] generally varied with study size. Similar to birth incidence, the highest prevalence estimate—one case per 264 children (aged 6 years old)—was from a small school-based study in Italy [22]. By comparison, three surveillance studies (from Cuba [21], Germany [17], and Israel [13]) with sample sizes between 19,404 and 152,819 children, and a population-based registry study (from Finland [14]), found lower estimates: from one case per 960 individuals [13] to one per 2996 individuals [17]. When examining prevalence by country, the two studies reporting the highest prevalence overall were the school-based study (from Italy) [22] and a review of military recruits (aged 17 years, from Israel) [14]. The lowest prevalence estimate of one per 5681 children was from a large database study in Northern Ireland that identified 75 NF-1 patients referred to a medical genetics center for evaluation, among the overall population of 425,250 individuals aged under 16 years [18].

Estimates of the prevalence of PNs in children with NF-1 from seven available studies [10, 15, 16, 18, 20, 21, 23] ranged from 0 [21] to 29.6% [23]. The variation in estimates may be due to study heterogeneity, in particular, difference in sample sizes. The two studies [15, 21] reporting the lowest prevalence, 0% and 5.7%, enrolled only 17 and 53 patients, respectively. Among studies with larger sample sizes (over 150 patients), and hence potentially greater reliability and generalizability, the prevalence of PNs ranged from 9.04 to 29.6% [10, 16, 23]. A trend toward higher prevalence in older populations was observed in one study, which reported that PNs affected 10.3%, 22.9%, and 27.8% of patients aged 0 to 10 years, 10 to 20 years, and > 20 years, respectively [20].

Evidence from two studies indicated that PNs carry an increased mortality risk in pediatric NF-1 [16, 23]. A prospective study of 405 children (aged < 17 years) with NF-1, conducted in North America, compared the clinical features of those that died during follow-up (mean duration 2.4 years) and those that survived and found that facial PNs were a significant mortality risk factor according to univariate analysis (reported p-value = 0.05) [16]. Similarly, a US retrospective study reported that, compared to patients with NF-1 without PNs or with asymptomatic, undetected PNs, those with symptomatic PNs had a higher mortality rate (3.2% vs. 0.5%, p = 0.024), over an average follow-up of 6.4 years [23]. However, no specific definition of “symptomatic” was given in the study report.

### Burden of pain

Among the studies examining baseline PN-related complications, pain was the most consistently reported problem [5, 25, 28, 32, 33]. Pain-related outcomes were reported in six unique studies: three retrospective analyses [23, 25, 26], two cross-sectional analyses [28, 31], and the phase II selumetinib trial (NCT01362803) [27, 29, 30]. Study sample sizes ranged from 50 [29, 30, 32] to 520 patients [23]. The pain outcomes reported varied across publications, covering, for example, the proportion of patients with PNs who experienced pain, pain reported as an indication for surgery, pain intensity (measured using various scales), and pain management. Collectively, the studies indicated that PN-related pain is common, often considerable, and potentially difficult to manage.

Five studies examined the proportion of patients with NF-1-associated PNs who experienced pain or how commonly PN-related pain was an indication for surgery [23, 25, 26, 28, 30]. One observational study found 67.1% of patients had PN-related pain [28]. The selumetinib trial reported that 70% of patients had pain at baseline [30]. Also, three studies reporting the proportion of PNs for which pain was an indication for subsequent surgery found this was 9.5% (16/168) of tumors in one study [25], over 18.8% (≥ 18/96 patients) in another [23], and 100% in a retrospective study of only 16 lesions operated on in a single institution [26].

Pain intensity was reported in three studies, each evaluating this outcome with a different tool. One study assessed pain interference using the Impact of Pediatric Illness (IPI) scale [31] for patients aged 6–18 years (by caregiver proxy rating) and adolescents aged 10–18 years (self-rating). It found the following results for these two groups: “no pain” (27% and 41%, respectively), “little pain” (22% and 22%), “some pain” (34% and 22%), “much pain” (15% and 10%), and “a lot of pain” (2% and 5%), respectively. Similarly, another study reported that 31% of pediatric patients had mild, 24% moderate, and 15% severe tumor pain, although it was unclear which scale was used to gather these data [30]. The third study reported that pain intensity among patients [28] assessed using the Patient-Reported Outcomes Measurement Information System (PROMIS; mean ± standard deviation: 49.75 ± 13.4) was not significantly different from a normative sample (50 ± 10). However, 67% of the children in this study reported pain, and the authors also noted considerable variation in the pain interference scores and suggested the substantial pain interference some patients experienced was “averaged out” by results for those with no pain.

Only one identified study assessed management of PN-related pain [31]. In this retrospective, US-based analysis, 33% of patients reported regular pain medication use: 3% using over-the-counter (OTC) medication (acetaminophen, ibuprofen) and 30% taking prescription medication (with/without OTC medication). Among those taking prescribed treatments, 61% reported being on anticonvulsants, 39% opioids, 33% antidepressants, and 17% using topical/ local anesthetics [31].

### Burden of surgery

Evidence on the clinical outcomes associated with surgery for PNs was reported by four studies [23–26]. Each of these involved reviewing medical records from a single institution in the US and had small sample sizes (11 [24] to 154 patients [23]). Pain was frequently reported as a reason for surgery [23–26], and other common indications included neurological deficits [23], loss of functionality [24], airway compression [23], and physical disfigurement or cosmetic reasons [23, 25, 26]. Minimal detail of surgical procedures and target PNs was reported, with a lack of information on such characteristics as the nature of PNs, their size, and their degree of vascularization. Data across the four studies indicated that surgery carries considerable risks, including high chances of tumor recurrence [23–26], even following complete excision [25].

All four studies reported on operation rates. The largest study (n = 154) provided rates for patients with NF-1 with associated PNs aged between 0 and 12 years [23]. Among these individuals, 62.3% with PNs underwent surgery during an average follow-up of 65.5 months. The three smaller studies reported operation rates per lesion, which ranged from 1.3 to 1.8 surgeries [23, 25, 26]. The studies did not report on whether there was any association between age and operation rates. One study reported the extent of resection achieved during surgery, as follows: gross total resection (15%), near-total resection (23%), and subtotal resection (44%) [25]. Secondary surgery rates were 43% of patients and 44% of lesions, as reported in two studies [23, 33].

Three of the studies on surgery also analyzed rates of post-operative complications [23, 25, 26], which ranged from 4.6% [25] of patients experiencing permanent neurological deficit to 18.8% [26] with wound-healing complications. Tumor recurrence (assessed in three studies) was also common, with the rate depending on the extent of tumor excision achieved. Specifically, recurrence rates across two studies [23, 25] ranged from 54.9 to 67.7% for partially resected tumors (< 50% excision); 28.8% to 44.6% following subtotal resections (defined as 50% to 80% or 90% excision); 39.5% following near-total resections (> 90% removal); and 20% for completely excised tumors. The third study involved only 11 patients and reported an 18% recurrence rate [24].

## Discussion

The evidence collated in this SLR demonstrates that children with NF-1-associated PNs experience substantial disease burden with significant unmet needs. It is important to note, however, the volume of identified literature on NF-1-associated PNs was limited, perhaps suggesting a lack of awareness and understanding of the condition in general and of the particular problems faced by patients with NF-1-associated PNs.

In general, the available prevalence estimates indicate that pediatric NF-1 is rare. However, there was considerable variation in the data across studies. The identified epidemiology studies were heterogeneous with regard to key characteristics, including variation in sample sizes (and therefore, possibly, the generalizability of any findings), differences in the age groups studied [34], and study procedures (e.g., case detection methods). Some evidence also suggested trends toward lower rates in northern European countries [14, 17, 18] than in the other regions for which data are available, although the evidence for this trend is very limited.

Similarly, clinical studies have reported wide-ranging estimates of the prevalence of PNs in NF-1, reaching up to 50%. However, the real-world studies identified in the review suggest that the prevalence of NF-1-associated PNs ranged between 0 and 30%. Definitive conclusions for the discrepancy between estimates from real-world analyses and those from other study types cannot be drawn on the data available. However, detection of internal or asymptomatic PNs through imaging modalities may have contributed to the higher estimates seen in clinical interventional studies. Additionally, higher prevalence estimates are likely to be reported in studies specifically focused on the identification of PNs. Furthermore, disease severity may be worse in patients evaluated in clinical studies or might differ due to study selection criteria, such that their results may not be generalizable to real-world populations.

The identified literature shows that PNs are often painful, with only a minority of patients (typically under 30%) reporting not having this symptom [30, 31]. Several studies evaluated the intensity of pain associated with PNs [28, 30, 31]; however, each used a different instrument, suggesting a lack of consensus on the most appropriate tool to assess PN-related pain in this population. Accordingly, the use of medication [31] for pain relief for these tumors is common and sometimes involves opioid treatment. Surgical resection of tumors is often performed for painful PNs but data in this review indicate considerable limitations of such therapy.

Details on the surgical procedures undertaken were inconsistently reported across the included studies. Nevertheless, it is clear from the available data that surgery carries a high risk of post-operative problems, including complications such as poor wound healing [26]. Furthermore, tumors may be unamenable to complete surgical excision due to their extent and/or location [25]. Also, recurrence is very common post-surgery [23–25], with even total excision of PNs being associated with around a 20% chance of this outcome [25], often resulting in the need for subsequent operations [23, 25, 26]. The evidence identified provided no clear insights into whether the rate of recurrence was associated with age.

### Strengths and limitations

The SLR was informed by the quality standards in the PRISMA guidelines [35] so it included clear documentation of the review methodology, search strategy and yields, and study attrition. Study selection criteria targeted publications that would best address the pre-defined research objectives and questions.

To our knowledge, no previously published SLR has investigated the epidemiology of NF-1 or systematically analyzed data on the burden of pain or surgical outcomes related to NF-1 with PNs. Therefore, this study addressed an evidence gap, using a systematic approach to synthesize the available evidence on these topics. It highlights the limited availability and heterogeneity of evidence on epidemiological outcomes and the lack of high-quality data on the burden of pain and surgery associated with PNs.

Most of the available evidence for these topics was retrospective, which increases its risk-of-bias compared with data generated by prospective study designs [36]. For some topics, the review relied on subgroup data reporting outcomes for the population of interest. Often, this meant that the primary objectives and overall conclusions of the individual studies were not specific to the focus of this SLR. The limited number of epidemiology estimates and the lack of global data on the burden of pain from real-world settings specifically highlight the need for further research on these topics.

## Conclusion

The identified evidence indicates that while NF-1 is a rare disease, up to around one-third of patients have PNs, and that these tumors can cause high morbidity. Surgical excision or resection of PNs carries considerable risk and is associated with a high rate of tumor recurrence. Overall, these findings suggest a need for better management options to minimize the disease burden in patients with NF-1-associated PNs.

## Supplementary Information

Below is the link to the electronic supplementary material.

Supplementary file1 (PDF 135 KB)

## Data Availability

Not applicable.

## Declarations

### Disclaimer

All named authors meet the International Committee of Medical Journal Editors (ICMJE) criteria for authorship for this manuscript, take responsibility for the integrity of the work as a whole, and have given final approval to the version to be published.
