# Supplementary material for: Epidemiological and clinical burden associated with plexiform neurofibromas in pediatric neurofibromatosis type-1 (NF-1): a systematic literature review
Source: Neurol Sci. 2021 Jun 18;43(2):1281–93. doi: 10.1007/s10072-021-05361-5 (PMC8789731; doi:10.1007/s10072-021-05361-5)
Supplement: Supplementary file 1 — Supplementary file1 (PDF 135 KB) [file 10072_2021_5361_MOESM1_ESM.pdf]

Epidemiological and Clinical Burden Associated with Plexiform Neurofibromas in Pediatric Neurofibromatosis Type-1 (NF-1): a Systematic Literature Review

Journal: Neurological Sciences

<u>Authors and Institutions:</u>

Ike Iheanacho, MB BS, BSc, Evidera, ike.iheanacho@evidera.com

Hyun Kyoo Yoo, MPH, AstraZeneca, HyunKyoo.Yoo@AstraZeneca.com

Xiaoqin Yang, PhD, Merck & Co., Inc., Kenilworth, NJ, USA, xiaoqin.yang@merck.com

Sophie Dodman, BSc, Evidera, sophie.dodman@evidera.com

Rachel Hughes, BA, Evidera, rachel.hughes@evidera.com

Suvina Amin, MPH, suvina.amin@astrazeneca.com

<u>Address for Correspondence:</u>

Ike Iheanacho, MB BS, BSc, Evidence, Modeling and Communication | Evidera

Address: The Ark, 201 Talgarth Rd, London, W6 8BJ, United Kingdom

Office: +44 (0) 208-576-500 | E-mail: ike.iheanacho@evidera.com

ONLINE SUPPLEMENT

Table S1 Embase via Ovid (October 2, 2019)

	Query	Yield
1	(neurofibromatosis type 1 or type 1 neurofibromatosis).ti,ab.	6,562
2	"NF1".ti,ab.	8,071
3	(Recklinghausen* or Recklinghausen* disease or Recklinghausen* syndrome or von Recklinghausen*).ti,ab.	3,043
4	1 or 2 or 3 or exp neurofibromatosis type 1/	14,208
5	plexiform neurofibroma*.ti,ab.	1,405
6	(epidemiology or incidence or prevalence or mortality or survival).ti,ab. or exp epidemiology/	5,129,940
7	(surgery or surg*).ti,ab. or exp surgery/	5,249,152
8	5 and 7	576
9	4 or 5	14,674
10	6 and 9	2,953
11	(pain*).ti,ab. or pain/ or pain assessment/ or pain measurement/	1,038,720
12	5 and 11	206
13	8 or 10 or 12	3,508
14	review.pt. not (systematic or meta$).mp.	1,875,541
15	(book or chapter or editorial or erratum or letter or note or short survey).pt.	3,107,781
16	exp quality control/ or "quality control".mp. or theoretical study/ or "theoretical study".mp. or methodology/ or "methodology study".mp.	2,440,552
17	animal cell/ or "animal cell".mp. or animal experiment/ or "animal experiment".mp. or animal model/ or "animal model".mp. or cancer cell culture/ or "cancer cell culture".mp. or human cell/ or "human cell".mp. or human tissue/ or "human tissue".mp. or exp in vitro study/ or "in vitro study".mp. or nonhuman/ or "nonhuman".mp. or exp biological model/ or "biological model".mp. or exp cell culture/ or "cell culture".mp. or diagnostic test accuracy study/ or "diagnostic test accuracy study".mp.	9,457,079
18	conference abstract.pt.	3,594,567
19	14 or 15 or 16 or 17 or 18	17,935,042
20	13 not 19	1,351

Table S2 MEDLINE via Ovid (October 2, 2019)

	Query	Yield
1	(neurofibromatosis type 1 or type 1 neurofibromatosis).ti,ab.	5,075
2	"NF1".ti,ab.	5,238
3	(Recklinghausen* or Recklinghausen* disease or Recklinghausen* syndrome or von Recklinghausen*).ti,ab.	3,214
4	1 or 2 or 3 or exp Neurofibromatosis 1/	14,088
5	plexiform neurofibroma*.ti,ab.	1,110
6	(epidemiology or incidence or prevalence or mortality or survival).ti,ab. or exp epidemiology/	2,619,572
7	(surgery or surg*).ti,ab. or exp General Surgery/	1,833,522
8	5 and 7	263
9	4 or 5	14,387
10	6 and 9	1,397
11	pain*.ti,ab. or exp Pain/ or exp Pain Measurement/ or exp Pain Management/	815,691
12	5 and 11	132
13	8 or 10 or 12	1,703
14	review.pt. not (systematic or meta$).mp.	1,695,649
15	(book or chapter or editorial or erratum or letter or note or short survey).pt.	1,547,298
16	exp quality control/ or "quality control".mp. or models, theoretical/ or "theoretical study".mp. or "methodology study".mp.	233,287
17	"animal cell".mp. or animal experimentation/ or "animal experiment".mp. or models, animal/ or "animal model".mp. or tumor cells cultured/ or "cancer cell culture".mp. or "human cell".mp. or tissues/ or "human tissue".mp. or exp in vitro techniques/ or "in vitro study".mp. or animals/ or "nonhuman".mp. or exp models, biological/ or "biological model".mp. or exp cell culture techniques/ or "cell culture".mp. or "diagnostic test accuracy study".mp.	7,074,312
18	14 or 15 or 16 or 17	9,982,885
19	13 not 18	1,337

Table S3 List of conferences included in grey literature searches

**Conference Searches**
American Society of Clinical Oncology (ASCO)
European Society for Medical Oncology (ESMO)
International Conference on Neurofibromatosis 1, Neurofibromatosis 2, and Schwannomatosis (Children's Tumor Foundation)
International Symposium on Pediatric Neuro-Oncology (ISPNO)
Society for Neuro-Oncology's Pediatric Neuro-Oncology Basic and Translational Research Conference.
